# Serum magnesium levels during magnesium sulfate infusion at 1 gram/hour versus 2 grams/hour as a maintenance dose to prevent eclampsia in women with severe preeclampsia: A randomized clinical trial

**DOI:** 10.1097/MD.0000000000016779

**Published:** 2019-08-09

**Authors:** Ana C. F. Pascoal, Leila Katz, Marcela H. Pinto, Carina A. Santos, Luana C. O. Braga, Sabina B. Maia, Melania M. R. Amorim

**Affiliations:** Instituto de Medicina Integral Prof. Fernando Figueira (IMIP), Recife, Pernambuco, Brazil.

**Keywords:** controlled clinical trials, dose-response relationship, magnesium sulfate, magnesium, preeclampsia

## Abstract

**Background:**

Magnesium sulfate is the ideal drug for the prevention and treatment of eclampsia. Nevertheless, the best regimen for protection against eclampsia with minimal side effects remains to be established. This study aimed to compare serum magnesium levels during intravenous infusion of magnesium sulfate at 1 gram/hour versus 2 grams/hour as a maintenance dose to prevent eclampsia in pregnant and postpartum women with severe preeclampsia.

**Methods:**

A randomized, triple-blind clinical trial was conducted, comparing serum magnesium levels during the intravenous infusion of magnesium sulfate at 1 gram/hour versus 2 grams/hour as a maintenance dose for the prevention of eclampsia in 62 pregnant and postpartum women with severe preeclampsia, 31 in each group. An intravenous loading dose of 6 grams of magnesium sulfate was administered over 30 minutes in both groups. The patients were then randomized to receive a maintenance dose of either 1 or 2 grams/hour for 24 hours. Primary outcomes consisted of serum magnesium levels at the following time points: baseline, 30 minutes, every 2 hours until the end of the first 6 hours, and every 6 hours thereafter until the termination of magnesium sulfate infusion. Side effects, maternal complications, and neonatal outcomes were the secondary outcomes.

**Results:**

Serum magnesium levels were higher in the 2-gram/hour group, with a statistically significant difference from 2 hours after the beginning of the magnesium sulfate infusion (*P* <.05). Oliguria was the most common complication recorded in both groups, with no significant difference between the 2 regimens (RR 0.88; 95% CI: 0.49–1.56; *P* = .65). No cases of eclampsia occurred. Side effects were more common in the 2-gram/hour group (RR 1.89; 95% CI: 1.04–3.41; *P* = .02); however, all were mild. There were no differences between the 2 groups regarding neonatal outcomes, except for admission to neonatal intensive care, which was more frequent in the 1-gram/hour group (25% vs 6.3%; *P* = .04).

**Conclusion:**

Magnesium sulfate therapy at the maintenance dose of 1 gram/hour was just as effective as the 2-gram maintenance dose, with fewer side effects.

## 1 Introduction

Preeclampsia/eclampsia occurs in 2% to 8% of pregnancies^[1,2]^ and is responsible for the death of 63,000 women worldwide every year. Around 9% of these deaths occur in Asia and Africa and 25% in Latin America and the Caribbean.^[1,3–6]^ In a multicenter study conducted in Brazil, the prevalence of severe maternal outcome (death or *near miss*) resulting from eclampsia was 5 times greater than that resulting from other severe complications related to hypertensive disorders of pregnancy. Eclampsia, therefore, remains the major cause of maternal morbidity and mortality, representing one of the principal reasons for admission to intensive care units (ICU).^[7]^

Preeclampsia is defined as the onset of hypertension associated with proteinuria or organ dysfunction after 20 weeks of pregnancy in women whose blood pressure was previously normal.^[8]^ Although little is known on the etiopathogenesis of preeclampsia, there is evidence that placental dysfunction occurs, with subsequent hypoperfusion of the uteroplacental bed.^[9,10]^ The final outcome is generalized arteriolar spasms, which, if the brain is affected, can lead to the onset of eclampsia, characterized by tonic-clonic self-limiting generalized seizures not attributable to any other cause.^[10,11]^

Magnesium sulfate is the ideal drug for the prevention and treatment of eclampsia,^[12,13]^ and, indeed, its universal use is recommended by the World Health Organization.^[14]^ Nevertheless, the best regimen remains to be established^[13]^ and there is still no evidence that serum magnesium levels between 4 and 7 mEq/L, established in a retrospective study and still considered therapeutic, represent a guarantee that pregnant women with hypertensive disorders are protected against eclampsia.^[15]^ Depending on the magnesium levels reached, side effects may be less common or more common^[16]^; therefore, the ideal regimen of magnesium sulfate should be a dose that protects against eclampsia with minimal side effects.

Initially, magnesium sulfate was administered intramuscularly, intravenously or subcutaneously, at a low dose. After the side effects of this treatment had been established, higher doses and different regimens were proposed.^[13]^ In 1955, Pritchard recommended an intramuscular regimen following an initial intravenous loading dose^[15]^; however, due to the pain associated with the injection and the possibility of local infection, the intravenous regimen gradually began to substitute intramuscular administration in the maintenance phase. Later, Zuspan proposed the intravenous infusion of 4 grams during the initial loading phase and 1 gram/hour as a maintenance dose,^[17]^ while in 1990, Sibai suggested using an initial loading dose of 6 grams followed by a maintenance dose of 2 grams/hour, both administered intravenously.^[18]^

Although the efficacy of magnesium sulfate for the prevention and treatment of eclamptic seizures has already been established, the best therapeutic regimen and the ideal duration of maintenance therapy have yet to be clarified. Therefore, the objective of the present study was to compare serum magnesium levels during the intravenous infusion of magnesium sulfate at 1 gram/hour versus 2 grams/hour as a maintenance dose for the prevention of eclampsia in pregnant and postpartum women with severe preeclampsia.

## 2 Methods

### 2.1 Study design

A randomized, triple-blind clinical trial was conducted, comparing serum magnesium levels during the intravenous infusion of magnesium sulfate at 1 gram/hour versus 2 grams/hour as a maintenance dose for the prevention of eclampsia in pregnant and postpartum women with severe preeclampsia. The study was conducted at the *Instituto de Medicina Integral Prof. Fernando Figueira* (IMIP) in Recife, Pernambuco, northeastern Brazil, between March 2015 and March 2016.

### 2.2 Patients and eligibility criteria

Pregnant women with severe preeclampsia, as defined by the American College of Obstetricians and Gynecologists (ACOG) diagnosis criteria,^[8]^ and submitted to treatment with magnesium sulfate at IMIP were included in this study. The ACOG criteria for diagnosis of severe preeclampsia are based on the presence of *any one* of the following findings in women with preeclampsia: systolic blood pressure ≥160 mmHg and/or diastolic blood pressure ≥110 mmHg, thrombocytopenia (<100,000/mm^3^), abnormal liver function (increase in transaminases to twice the normal value or pain in the upper right quadrant/epigastrium that is unresponsive to medication), abnormal renal function (creatinine >1.1 mg/dl or twice normal values), acute pulmonary edema and/or new cerebral and/or visual symptoms.

The exclusion criteria consisted of the occurrence of eclampsia prior to administration of the initial loading dose of magnesium sulfate; use of other medicines or illicit drugs that could interfere with maternal hemodynamics, contraindications to the use of magnesium sulfate (known hypersensitivity to the drug, oliguria with urinary output below 25 mL per hour, or severe myasthenia), acute or chronic kidney disease and a diminished level of consciousness.

### 2.3 Interventions

All the patients selected for the study were submitted to the standard loading dose of 6 grams of magnesium sulfate administered over 30 minutes, in compliance with this institute's guidelines.^[19]^ After signing an informed consent form and having received the initial loading infusion dose, the patients were randomized to receive a maintenance dose of magnesium sulfate of 1 gram/hour or 2 grams/hour.

### 2.4 Assessments and outcomes

The following baseline variables were analyzed and compared to ensure that there were no statistically significant differences between the groups at admission: maternal age, gestational age, parity, blood pressure, creatinine, symptoms of imminent eclampsia, and the presence of associated comorbidities such as hyperthyroidism/hypothyroidism, cardiovascular diseases, renal failure, systemic lupus erythematosus, antiphospholipid antibody syndrome, liver disease, diabetes, and obesity.

The primary outcomes consisted of the levels of serum magnesium at the following time points: baseline, 30 minutes, every 2 hours until the end of the first 6 hours, and every 6 hours thereafter until termination of the magnesium sulfate infusion. The effectiveness in reaching serum magnesium levels considered therapeutic as a function of time was also evaluated.

The secondary outcomes analyzed were: the occurrence of maternal complications (eclampsia, placental abruption, postpartum hemorrhage, retained placenta, thromboembolic complications, liver failure, oliguria, kidney failure, disseminated intravascular coagulation, cerebrovascular accident, and acute pulmonary edema), side effects (heat sensation, facial redness, somnolence, confusion, dizziness, thirst, muscle weakness, headache, hypersensitive reaction, nausea, and vomiting), treatment discontinuation due to side effects, need for calcium gluconate, hypertensive peaks, mode of delivery, and neonatal outcomes (respiratory disorders, need for resuscitation, need for assisted mechanical ventilation, need for ICU admission, and neonatal death).

### 2.5 Randomization

A randomization list was prepared using the Random Allocation software program, version 1.0. This list consisted of sequential numbers ranging from 1 to 2000 plus the letters “A” or “B” as originally programmed for a study entitled “Comparison of the effectiveness and safety of a maintenance dose of 1-gram/hour versus 2-grams/hour infusions of magnesium sulfate for the prevention of eclampsia in women with severe preeclampsia: a randomized clinical trial.” This list was divided into blocks of 20. During the data collection period, it was noted that the recruitment rate was lower than expected. Therefore, in order to analyze serum magnesium in patients submitted to both magnesium sulfate regimens, it was decided to suspend the initially proposed study, remove the blinding and analyze the 62 patients according to the new calculation of sample size.

### 2.6 Blinding

The pharmacist received the randomization list of numbers from 1 to 2000, with the letters “A” or “B”, defining whether the patient would be in the 1-gram/hour or 2-grams/hour group. The pharmacist then prepared ampoules with distilled water for the 1-gram/hour group, and ampoules with a total of 6 grams of magnesium sulfate for the 2-grams/hour group. Both sets of ampoules were identical in color and size. Only the pharmacist was aware of the contents of the ampoules. The numbered envelopes were sent to the high-risk unit and to the intensive care unit where the women were consecutively assigned to one of the maintenance regimens. The numbered envelope containing the ampoules was only opened at the time of preparation of the maintenance dose of magnesium sulfate.

### 2.7 Sample size calculation

Sample size for this study was calculated using the publicly available OpenEpi software program, version 3.01, taking into consideration the data from a study published in 2013 in which 60% of the women receiving 2 grams/hour of magnesium sulfate as a maintenance dose achieved magnesium levels within the therapeutic range within 2 hours postpartum compared to 20% of those who received a dose of 1 gram/hour.^[20]^ To detect this difference for a power of 80% (Type II [beta] error) and a bilateral significance level of 95% (Type I [alpha] error), according to Fleiss's formula with continuity correction factor, 56 patients would be required: 28 in each group. This number was increased to 62 patients to compensate for any losses.

### 2.8 Treatments

Two groups of patients were then formed, based on the following possible regimens for the maintenance therapy:

The 1 gram/hour of magnesium sulfate: The maintenance dose was initially prepared by diluting 12 mL of 50% magnesium sulfate in 476 mL of 0.9% saline solution. An ampoule containing 12 mL of distilled water taken from the randomization envelope (in accordance with the sequential number assigned to each individual patient) was then added to the infusion solution. The speed of infusion was 84 mL/hour, resulting in a regimen of 1 gram/hour.

The 2 grams/hour of magnesium sulfate: The maintenance dose was initially prepared by diluting 12 mL of 50% magnesium sulfate in 476 mL of 0.9% saline solution, as described above. An ampoule containing 12 mL of magnesium sulfate taken from the randomization envelope (in accordance with the sequential number assigned to each individual patient) was then added to the infusion solution. The speed of infusion was 84 mL/hour, resulting in a regimen of 2 grams/hour.

Throughout the entire study, the investigators, the attending physicians, and the patients remained unaware of the group to which the patient had been allocated (1 gram/hour or 2 grams/hour).

From the beginning of the magnesium sulfate infusion for the maintenance phase, blood samples were taken using a saline flushed intravenous catheter placed in the opposite arm to that used for the infusion. This enabled serial analysis to be performed of serum magnesium levels at different time points (baseline, 30 minutes, every 2 hours for the first 6 hours and every 6 hours thereafter). The patients’ heart rate, respiratory rate, diuresis, blood pressure, and deep reflexes were evaluated every 6 hours by their attending physician and by the investigators. The magnesium sulfate infusion was to be interrupted if respiratory rate fell to <12 breaths per minute and/or diuresis to <25 mL per hour and/or if deep reflexes were found to be diminished or absent, with the medication being reinitiated as soon as these adverse conditions were reverted. There are no guidelines determining when reflexes are diminished, normal or increased, with this being left to the examiner's discretion.^[21]^

### 2.9 Statistical analysis

Analysis was conducted using EpiInfo, version 7 (Atlanta, GA) and the Medcalc software program, version 15.6.1 (Medical Software bvba). The baseline characteristics of the patients in the 2 groups were compared using Student's *t* test for continuous variables with normal distribution. The Mann–Whitney test was used for discrete and ordinal variables or for continuous variables with non-normal distribution. The categorical variables were compared in contingency tables using Pearson chi-square test of association or Fisher exact test, as pertinent. To compare serum magnesium levels in the 2 groups, repeated measures analysis was performed, assuming sphericity. Graphs were generated, the first showing the curve of mean values (with 95% confidence intervals [95%CI]) for the various time points within each group and the second showing a dot and line diagram comparing all the values at each time point per group. All *P* values were 2-tailed, and the significance level adopted was 5%.

### 2.10 Ethical issues

The institution's internal review board approved this study under reference number 37560214.0.0000.5201 and the protocol was registered at ClinicalTrials.gov (www.clinicaltrials.gov) under reference number NCT02396030.

## 3 Results

**Figure 1:**
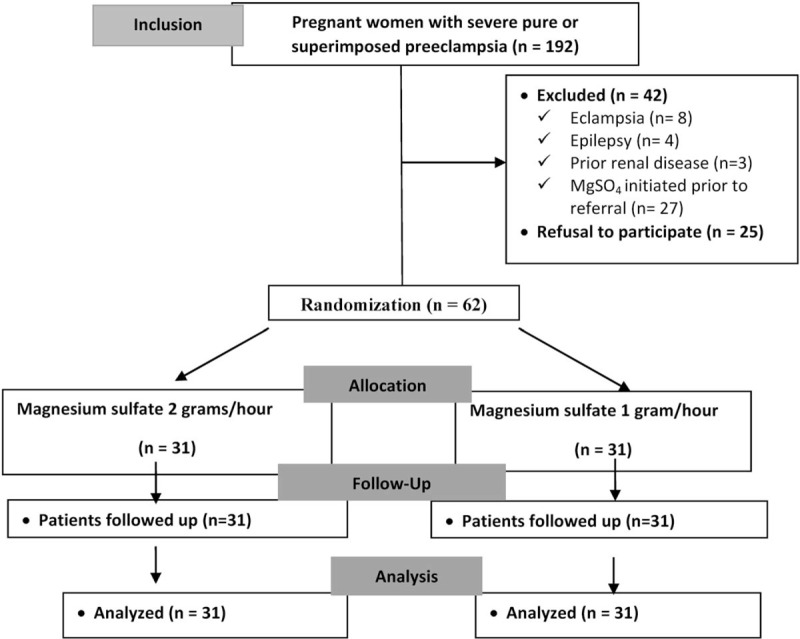
Procedures for the selection and follow up of participants (CONSORT flowchart).

During the study period, 192 patients with severe preeclampsia were admitted and 129 of these patients were approached by the study team. Of these, eight women were excluded because of eclampsia, 4 due to epilepsy, 3 because of prior renal disease and 27 because they had received the initial loading dose of magnesium sulfate in another hospital prior to their transfer to this institute. Another 25 patients refused to participate in the study. Therefore, a final population sample of 62 patients was included and randomized to the 1-gram (n= 31) or 2-gram group (n= 31). No discontinuations occurred in either of the groups (Fig. 1).

The baseline characteristics were similar in both groups (Table 1). Severe preeclampsia was more prevalent than severe superimposed preeclampsia in both groups (61.3% in the 2-gram/hour group and 64.5% in the 1-gram/hour group). HELLP syndrome developed in 12.9% of the patients in the 2-gram group and in 10.3% in the 1-gram group. The frequency of associated comorbidities was the same in both groups (32.3%).

**Table 1:**
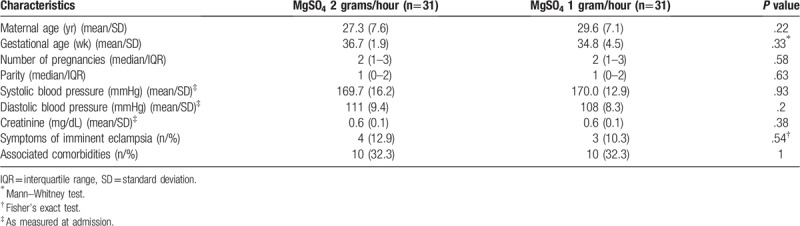
Baseline characteristics of women with severe preeclampsia. *Instituto de Medicina Integral Prof. Fernando Figueira*, Recife, Pernambuco, Brazil.

**Figure 2:**
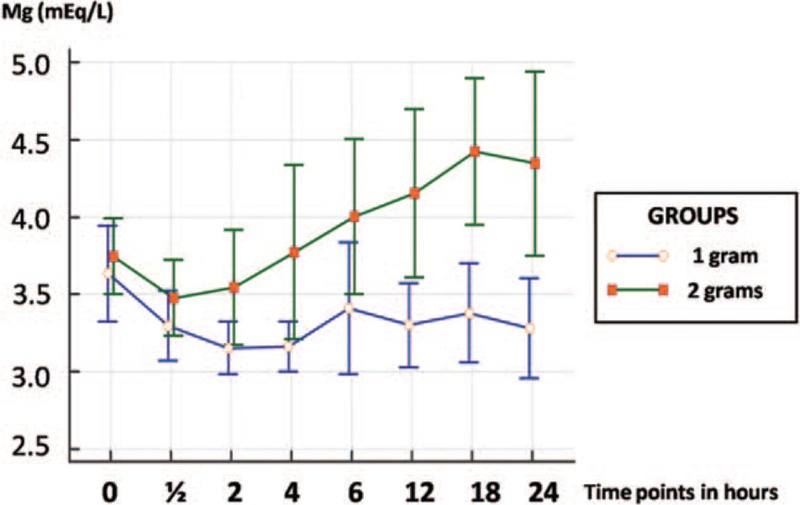
Curve of serum magnesium levels in mEq/L over the 24-hour magnesium sulfate infusion period showing the progressive, significant increase of mean magnesium levels in the 2-gram group at the end of the maintenance therapy, while concentration was more constant in the 1-gram group. Values are expressed as means and 95% confidence intervals at each point.

**Figure 3:**
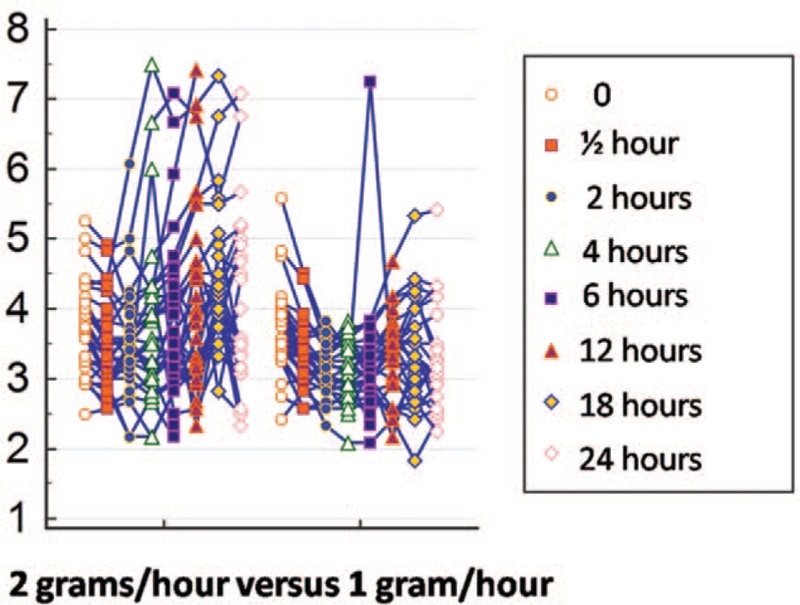
Curve of serum magnesium levels in mEq/L over 24 hours, individualized for each patient. Note the greater frequency of higher concentration spikes in the 2-gram group.

The levels of serum magnesium measured at the beginning of the maintenance phase and those measured 30 minutes later were not significantly different between the groups. Nevertheless, at the other time points, a statistically significant difference was found between the 2 groups, particularly at the end of the third and fourth 6-hour phases of the maintenance therapy, when magnesium levels were much higher in the 2-gram group (Table 2, Figs. 2 and 3).

**Table 2:**
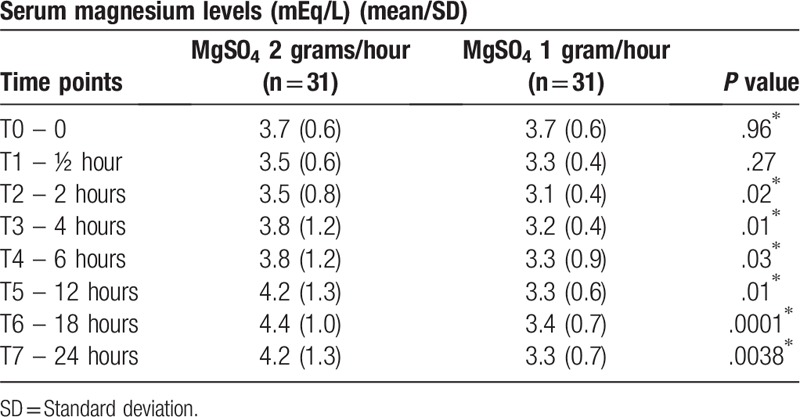
Magnesium levels during the 24 hours of maintenance therapy with MgSO_4_ infusion.

Oliguria was the most common complication recorded in both groups, with no significant difference between the 2 regimens. Only 1 patient in the 1-gram group developed kidney failure and another 2 patients in that same group suffered postpartum hemorrhage, which was rapidly resolved in both cases. Treatment had to be extended in 12.9% of the patients in the 2-gram group and in 6.9% in the 1-gram group, whereas treatment had to be reinitiated in more patients in the 1-gram group (10.3%) than in the 2-gram group (6.7%) (Table 3). There were no cases of placental abruption, disseminated intravascular coagulation, cerebrovascular accident, acute pulmonary edema, eclampsia, or death.

**Table 3:**
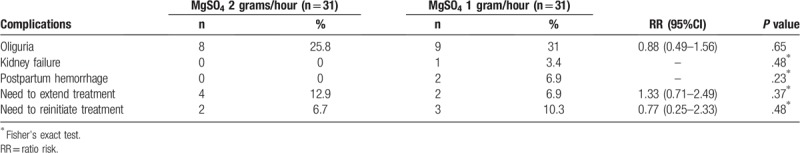
Maternal complications in women with severe preeclampsia. *Instituto de Medicina Integral Prof. Fernando Figueira*, Recife, Pernambuco, Brazil.

The incidence of hypertensive peaks was high and similar in both groups. The range of serum magnesium levels considered therapeutic (4–7 mEq/L) was achieved by only 5 patients submitted to the 2-gram/hour regimen. On the other hand, these levels were not achieved by any of the patients randomized to the 1-gram/hour regimen. The frequency of side effects was significantly greater in the 2-gram/hour group compared to the group using 1 gram/hour (71% versus 41.9%). The most common side effects in the 2-gram group were: heat sensation, nausea, and thirst (all occurring in 22.6% of patients). In the 1-gram group, the most common side effects were heat sensation (16.1%) and nausea (12.9%). In no cases did treatment have to be interrupted because of the onset of side effects, and calcium gluconate was not required in any of the cases (Table 4).

**Table 4:**
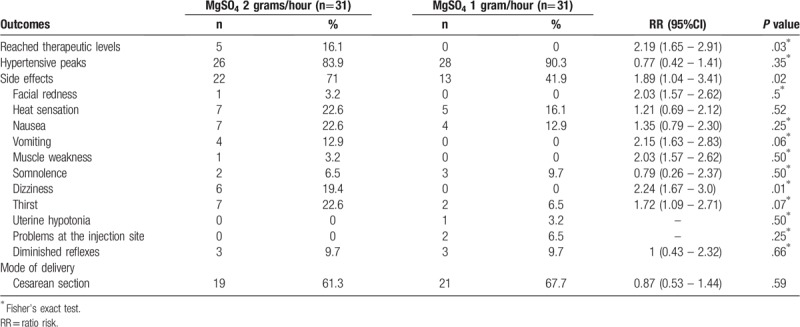
Maternal outcomes for women with severe preeclampsia submitted to magnesium sulfate therapy. *Instituto de Medicina Integral Prof. Fernando Figueira*, Recife, Pernambuco, Brazil.

Cesarean section was the most common mode of delivery; however, the incidence was similar in both groups: 61.3% of patients in the 2-gram group and 67.7% in the 1-gram group (Table 4).

Since 2 of the 62 women in the study had twins, data analysis was conducted on 64 newborn infants, with 2 of these babies having been born in a private hospital because the mothers were transferred before delivery. Mean gestational age was 36.8 ± 1.74 weeks (± SD) for the 2-gram group and 35.1 ± 4.58 weeks for the 1-gram group, with no statistically significant differences between the groups. There was a statistically significant difference in relation to birthweight, with an average weight of 2917 grams in the 2-gram group and of 2436 grams in the 1-gram group (*P* = .03). There were no statistically significant differences between the 2 groups in relation to 1st or 5th minute Apgar scores, or to neonatal outcomes, with the only difference being in the frequency of admission to the intensive care unit, which was higher in the 1-gram group (6.3% in the 2-gram group and 25% in the 1-gram group) (Table 5).

**Table 5:**
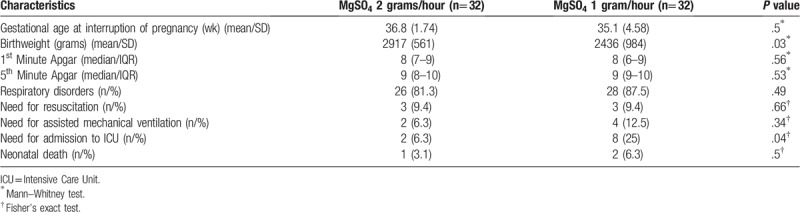
Characteristics of the newborn infants and neonatal outcomes.

## 4 Discussion

Serum magnesium levels were higher in the group submitted to the 2-gram/hour regimen compared to those of the women allocated to the 1-gram/hour regimen, except for the first 2 doses. The finding that there was no significant difference in mean magnesium levels at the first 2 measurements may have been a consequence of the similar initial loading dose in both groups, together with the fact that the drug had probably not yet reached the mean maximum plasma concentration, reflecting the stable state of dynamic equilibrium between the dose of the drug administered and its distribution and elimination rate.^[22]^ As expected, by the third measurement, magnesium levels were significantly higher in the group receiving the higher dose.

Of all the women analyzed, only 5 patients in the 2-gram/hour group achieved the magnesium levels considered in the literature to be therapeutic (between 4 and 7 mEq/L), as proposed in an observational study published in 1955.^[15]^ This number was well below expectations; however, outcome was not severe in any of the patients in the present study. Recently, a systematic review that included studies with various magnesium sulfate regimens found that, in the great majority of cases, serum magnesium levels were below the level considered therapeutic, although higher levels were also found with the 2-gram/hour regimen and with intramuscular administration. A considerable proportion of the studies analyzed involved a high risk of bias, and randomized clinical trials were a minority.^[23]^ The target level established as “therapeutic” may have been proposed without the availability of adequate studies to compare different regimens, without profound knowledge of the pharmacokinetics and pharmacodynamics of the drug, and without determining the association between magnesium levels and the effective prevention of eclamptic seizures.

One of the clinical trials included in the systematic review evaluated serum magnesium levels in only 17 patients, comparing an intramuscular regimen with an intravenous one.^[24]^ Although another randomized clinical trial evaluated a total of 194 women with preeclampsia submitted to the same dose of magnesium sulfate and divided into 4 groups according to their body mass index, magnesium levels were measured only twice, once before and once following delivery.^[25]^ In fact, the limitations with most of those studies referred not only to the small sample sizes and few blood samples collected per patient to evaluate magnesium levels, but also to the inclusion of patients with eclampsia.^[23]^

The results of the present study are similar to those summarized in the systematic review^[23]^ and highlight the need to reevaluate the serum levels of magnesium that should be achieved in order to avoid seizures. The present results suggest that this therapeutic level may be lower than that previously proposed by Pritchard.^[15]^

The multiple measurements of serum magnesium levels allowed 2 curves to be constructed, enabling the changes in levels to be monitored throughout the period of magnesium sulfate infusion. A study conducted in 1993 also took consecutive magnesium measurements in 44 women with preeclampsia; however, that was a prospective study that evaluated only 1 intravenous magnesium sulfate regimen.^[26]^ One of the objectives of a randomized pharmacokinetic study conducted in 2013 was also to construct a curve with magnesium level measurements; however, the comparison made was between an intravenous regimen (an initial loading dose of 4 grams and a maintenance dose of 1 gram/hour) and an intramuscular regimen (an initial loading dose of 4 grams administered intravenously in association with 10 grams administered intramuscularly and a maintenance dose of 5 grams every 4 hours).^[27]^ In summary, few studies have performed a curve analysis of serum magnesium levels, comparing 2 intravenous regimens using well-designed methodology.

The present findings showed that serum magnesium levels initially fell. This may have occurred as a consequence of the alpha or distribution phase, which corresponds to the distribution of the drug from the central compartment (blood) to the peripheral compartments (tissues).^[22]^ From then onwards, while the curve for the 2-gram/hour group rose, concentration was more constant in the 1-gram group, with magnesium levels remaining within the same range. In relation to the group exposed to the lower dose, the constant serum magnesium curve could be explained by the fact that the 1-gram/hour dose was probably sufficient for the constant mean maximum plasma concentration to be achieved, at which point a plateau is reached and there is practically no further variation in drug levels. The increasing values in the group in which the dose was higher may have been the result of saturation of the elimination mechanisms of the drug and, consequently, an accumulation in the body, perhaps leading to toxic effects.^[22]^

The rate of side effects was much higher in the 2-gram group and this difference was statistically significant. This finding is in agreement with reports in the literature affirming that these effects are directly associated with serum magnesium levels. Nevertheless, the side effects observed here were mild, and there was no need to discontinue treatment in any of the cases. The rate of side effects found in a Cochrane meta-analysis was much lower than that found in this randomized study (71% in the 2-gram group and 41.9% in the 1-gram group versus 24% in the meta-analysis); however, this difference may have occurred because in the present study this information was rigorously recorded, with even the mildest side effects being registered, whereas in the meta-analysis only major side effects were included.^[12]^ No severe adverse events that would have been indicative of the toxicity of the drug were observed in either of the 2 groups evaluated. These results are in agreement with other reports, including a systematic review that found a low incidence of drug toxicity (diminished patellar reflexes in 1.6% and respiratory depression in 1.3%).^[28]^

There were no cases of eclampsia in either of the 2 groups evaluated. This finding was expected, since previous publications have confirmed that eclampsia is a rare event in patients in use of anticonvulsants, with an incidence of 0.6%.^[29]^ Nevertheless, since the sample size of this trial was not calculated for this specific purpose, further studies are needed to evaluate this outcome with a larger sample size. Multicenter studies should be conducted to ensure that a sufficient number of patients are included within an adequate period of time.

The incidence of episodes of very high blood pressure was similar in both groups, which supports the hypothesis that magnesium sulfate has little effect on blood pressure levels even when used at different doses.^[23]^

Oliguria was the most common of the complications associated with preeclampsia; however, as with the other complications of the disease, the frequency was similar in both groups. The most common reason associated with a need to interrupt treatment was oliguria. Side effects did not result in a need to interrupt treatment in any of these cases.

The use of magnesium sulfate has also been associated with a 5% increase in the risk of Cesarean section compared to the use of placebo or of other anticonvulsants.^[12]^ In the present study, there was no statistically significant difference in the Cesarean section rate between the 2 groups. In the 1-gram group, 2 patients suffered postpartum hemorrhage compared to none in the 2-gram group. The effects of magnesium sulfate as a smooth muscle relaxant remain to be fully clarified; however, as in other studies published in the literature, the doses used in the present study did not appear to exert this effect; therefore, both dose regimens appear to be safe.

Since magnesium sulfate can cross the placental barrier, it may also act as a vasodilator and muscle relaxant in the neonate. Evidence already exists on its neuroprotective effect^[30–32]^; however, some studies have evaluated other consequences of exposure to this drug. A retrospective cohort study evaluated 6654 women submitted to treatment with magnesium sulfate, with a maintenance dose that ranged from 2 to 3 grams/hour. A greater incidence of hypotonia and of lower Apgar scores was found in the newborn infants, as well as a greater need for intubation in the delivery room and admission to the ICU. These adverse events were directly associated with serum magnesium levels, which ranged from 3 to 7 mEq/L.^[32]^ Another cross-sectional cohort study published in 2015 corroborated the hypotheses described above; however, it is important to emphasize that the dose of magnesium sulfate used was considerably higher than that used in this clinical trial, so much so that most of the women had magnesium levels between 4 and 7 mEq/L.^[3]^

In the present study, a difference was found in relation to birthweight. Although statistically significant, this difference is probably not clinically significant and may have been random as a consequence of the small sample size. In fact, despite the small sample size, there was no statistically significant difference between the 2 groups in relation to the neonatal outcomes analyzed, except for the frequency of admission to the intensive care unit, which was higher in the 1-gram group, probably because in most cases serum magnesium levels were below the level initially expected. The magnesium sulfate levels cannot explain this difference in the rates of admission to the intensive care unit, since most admissions occurred in patients with lower levels. A difference in the criteria for admission to an intensive care unit, particularly in the group in which the birthweight was lower, may explain this finding. A larger sample may be necessary to clarify this outcome.

It is reasonable to speculate that the adverse neonatal effects seen in women exposed to magnesium sulfate were directly proportional to the dose of the drug used and to the corresponding magnesium levels; however, there was no increase in the incidence of these effects in the newborn infants at either of the doses used in the present study.

The most important limiting factor of the present study was its small sample size, which prevents any inferences from being made regarding whether the 2 regimens used provide equal protection against eclampsia. Nevertheless, for this analysis, eclampsia was not the primary outcome. It should also be emphasized that this sample size was similar to or larger than those used in other published studies.

Despite the restricted sample size, the possibility of bias was reduced with this study design. Furthermore, simulation of the curves of the magnesium levels throughout the period of magnesium sulfate infusion, a factor that has been described in few studies, came close to real-life data and, with this, it was possible to rule out the hypothesis that magnesium levels would be higher in the 2-gram/hour group compared to the 1-gram control group. Contrary to what was expected, few patients achieved the magnesium levels that are considered therapeutic, although no patients suffered seizures. Although side effects were mild, they were more common in the 2-gram group.

These data are still preliminary and need to be confirmed in larger studies with a similar study design before any definitive conclusions can be reached; however, we believe that the 1 gram/hour magnesium sulfate maintenance regimen is preferable to the 2 gram/hour regimen for most patients with severe preeclampsia, since it exposes the patient to a lower dose of the drug and, consequently, to fewer side effects, without increasing the incidence of negative maternal or fetal outcomes.

Although a systematic review has already been published on the subject, we believe that further well-designed studies with larger sample sizes need to be conducted to enable a definitive decision to be reached regarding the best prophylactic regimen for such a detrimental event as eclampsia.

## Author contributions

**Conceptualization:** Ana C. F. Pascoal, Leila Katz, Sabina B. Maia, Melania M. R. Amorim

**Data curation:** Ana C. F. Pascoal, Marcela H. Pinto, Carina A. Santos, Luana C. O. Braga

**Formal analysis:** Ana C. F. Pascoal, Leila Katz, Melania M. R. Amorim

**Investigation:** Ana C. F. Pascoal, Marcela H. Pinto, Carina A. Santos, Luana C. O. Braga

**Methodology:** Ana C. F. Pascoal, Leila Katz, Sabina B. Maia, Melania M. R. Amorim

**Project administration:** Leila Katz, Melania M. R. Amorim

**Supervision:** Melania M. R. Amorim

**Writing – original draft:** Ana C. F. Pascoal, Leila Katz

**Writing – review & editing:** Sabina B. Maia, Melania M. R. Amorim
